# A late sharp increase in influenza detections and low interim vaccine effectiveness against the circulating A(H3N2) strain, Denmark, 2021/22 influenza season up to 25 March 2022

**DOI:** 10.2807/1560-7917.ES.2022.27.15.2200278

**Published:** 2022-04-14

**Authors:** Hanne-Dorthe Emborg, Lasse S Vestergaard, Amanda Bolt Botnen, Jens Nielsen, Tyra G Krause, Ramona Trebbien

**Affiliations:** 1 Statens Serum Institut, Copenhagen, Denmark

**Keywords:** Vaccine effectiveness, 2021/22 influenza season, Influenza A(H3N2)

## Abstract

We estimated interim influenza A vaccine effectiveness (VE) following a late sharp rise in cases during an influenza A(H3N2)-dominated 2021/22 season, after lifting COVID-19 restrictions. In children aged 2–6 years offered a live attenuated influenza vaccine, adjusted VE was 62.7% (95% CI: 10.9–84.4) in hospitalised and 64.2% (95% CI: 50.5–74.1) in non-hospitalised children. In non-hospitalised patients aged 7–44 years, VE was 24.8% (95% CI: 12.8–35.2); VE was non-significant in remaining age groups and hospital/non-hospital settings.

In week 12 2020, a range of interventions and restrictions to prevent the spread of severe acute respiratory syndrome coronavirus 2 (SARS-CoV-2) were introduced in Denmark, which led to a sharp decline in the influenza occurrence [1]. Since then, only sporadic laboratory-confirmed influenza cases have been detected. Here we present interim vaccine effectiveness (VE) estimates of seasonal influenza vaccines following the sharp increase in the influenza occurrence late in the season after the lifting of the COVID-19 restrictions in Denmark.

## Influenza detections in Denmark up to week 12 2022

From mid-February 2022, upon the relaxing of restrictions, the weekly number of cases doubled from 65 in week 6 to 3,178 in week 12 and, despite a high testing activity, the percentage of positive tests continued to increase (Figure). The circulating strain for the 2021/22 season is influenza A(H3N2) and mainly clade 3C.2a1b.2a.2, which differs from the vaccine strain (clade 3C.2a1b.2a.1). 

**Figure:**
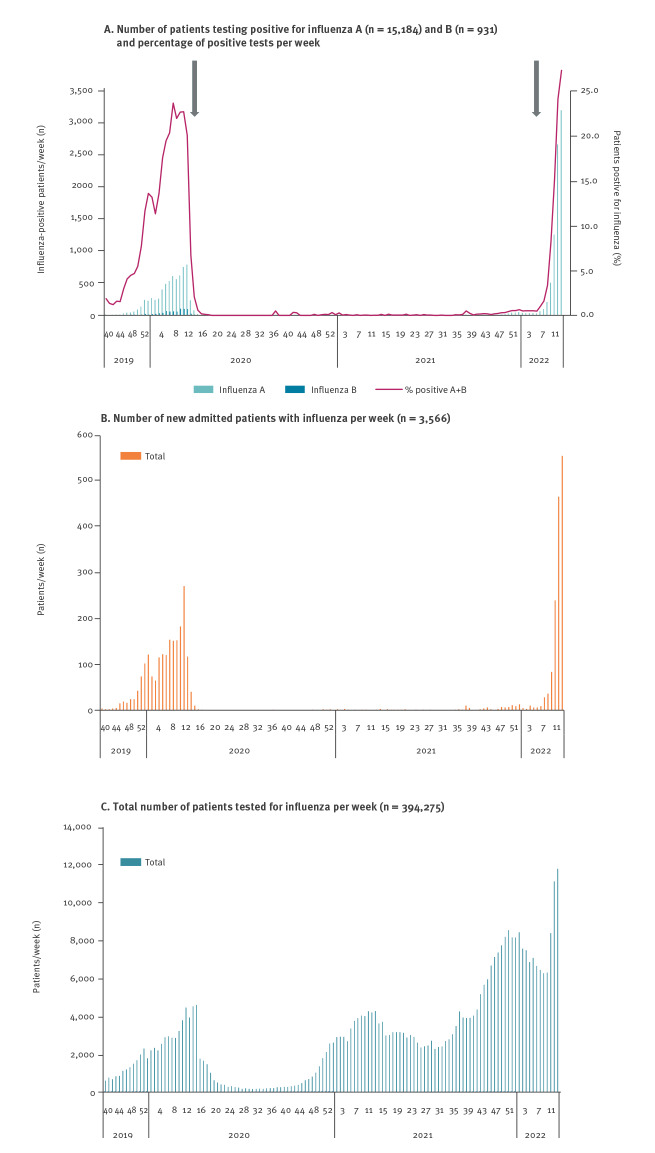
A. Number of patients testing positive for influenza A and B and percentage positive tests, B. number of new patients admitted to hospital with influenza and C. total number of patients tested for influenza by week, Denmark, week 40 2019–week 12 2022

In panel A, the grey arrows indicated points in time for implementation (left arrow) and lift (right arrow) of coronavirus disease (COVID-19) restrictions.

## National monitoring of laboratory-confirmed influenza cases and seasonal vaccination 

For the 2021/22 season, influenza vaccination was – in accordance with previous seasons – offered free of charge to all individuals aged 65 years and above, and to individuals with a risk of severe influenza. In addition, vaccination was also recommended and offered free of charge to healthcare workers and children aged 2–6 years. Children between 2–6 years of age were offered a live attenuated influenza vaccine (LAIV). The rest of the population was offered quadrivalent inactivated influenza (QIV) vaccines. Individuals were considered vaccinated if they had received one dose of the 2021/22 influenza vaccine at least 14 days before being tested for influenza, except children under 9 years of age; they should either receive two doses in the current season or one dose in the previous season and one in the current season at least 14 days before being tested for influenza. All administered influenza vaccines are registered in the Danish Vaccination Register (DVR) [2]. By the end of October 2021, 67% of the 2021/22 influenza vaccine doses were administered.

During the influenza season, national guidelines in Denmark recommend that patients belonging to risk groups, including elderly people who present with influenza-like illness (ILI) at a general practitioner (GP) or with ILI and/or lower respiratory symptoms at hospitals, are swabbed and tested for influenza virus (http://www.infmed.dk/guidelines). In the Danish Microbiology Database (MiBa), data on all patients swabbed at the GP or at hospitals and tested for influenza A and B viruses by PCR are registered in real-time [3]. Each sample result provides information on the date of sampling and if the sample was positive or negative for influenza virus. A random subsample of the positive influenza tests was subtyped and genetically characterised at the National Influenza Center at Statens Serum Institut.

Information on hospital admission and underlying disease(s) were available from the National Patient Registry (NPR) [4]. Patients swabbed by the GP were counted as non-hospitalised, patients swabbed within 4 days of or during an admission lasting less than 12 h in duration were also considered as non-hospitalised, while patients admitted for 12 h or more were considered as hospitalised. An increase in patients admitted to the hospital following infection with influenza A was observed, from nine patients in week 6 to 549 in week 12.

## Estimating vaccine effectiveness against the circulating influenza A strain in Denmark

To examine vaccine effectiveness (VE) we initially identified all individuals tested for influenza from week 40 in 2021 and onwards. Data from MiBa, DVR and NPR were linked using the unique person-identifier that all Danish citizens receive at birth or immigration.

We excluded individuals vaccinated less than 14 days before the date of swabbing, all children aged 0–1 year and swabs taken in October and November 2021, when no influenza virus was circulating. We used the test-negative case–control design. Cases were patients who were swabbed and tested positive for influenza A virus using real-time RT-PCR. Controls were patients who tested negative for any influenza virus using RT-PCR. The first test – either positive or negative – for each patient was included. We estimated the vaccine effectiveness (VE) as ((1 − odds ratio (OR) of vaccination between cases and controls) x 100) using logistic regression. All VE estimates were adjusted for calendar time by month of sampling and potential confounding factors included sex and underlying chronic conditions. We stratified VE by age groups.

Of the total 7,691 influenza A cases, 82.0% (n = 6,308) were detected in non-hospitalised patients and 63.1% (3,979/6,308) of these patients were 2–30 years of age, while 68.5% (947/1,383) of the hospitalised influenza A cases were detected in patients aged 45 years and older. In both hospitalised and non-hospitalised patients, females tested positive for influenza A more frequently. Of confirmed influenza A cases, underlying chronic conditions were found in 24.5% of those not hospitalised and in 65.0% of those hospitalised. Among non-hospitalised patients with influenza A, only 19.2% were vaccinated compared with 55.0% in hospitalised cases (Table 1).

**Table 1:** Influenza A cases and controls in 2021/22 interim seasonal influenza vaccine effectiveness analysis of non-hospitalised and hospitalised patients, Denmark, 1 December 2021–25 March 2022 (n = 89,183)

Variables	Non-hospitalised patients (n = 38,906)				Hospitalised patients (n = 50,277)			
	Influenza A cases		Controls		Influenza A cases		Controls	
	n	%	n	%	n	%	n	%
Total	6,308	16.2	32,598	83.8	1,383	2,7	48,894	97.3
Age group (years)								
2–6	473	7.5	1,692	5.2	43	3.1	869	1.8
7–17	1,923	30.5	1,953	6.0	139	10.1	1,151	2.4
18–30	1,583	25.1	5,858	18.0	147	10.6	4,040	8.3
31–44	835	13.2	5,808	17.8	107	7.7	4,121	8.4
45–64	877	13.9	8,448	25.9	210	15.2	9,755	20.0
≥ 65	617	9.8	8,839	27.1	737	53.3	28,958	59.2
Total	6,308	100.0	32,598	100.0	1,383	100.0	48,894	100.0
Sex								
Female	3,401	53.9	18,289	56.1	697	50.4	24,940	51.0
Male	2,907	46.1	14,309	43.9	686	49.6	23,954	49.0
Total	6,308	100.0	32,598	100.0	1,383	100.0	48,894	100.0
Chronic conditions^a^								
Yes	1,547	24.5	13,509	41.4	899	65.0	31,500	64.4
No	4,761	75.5	19,089	58.6	484	35.0	17,394	35.6
Total	6,308	100.0	32,598	100.0	1,383	100.0	48,894	100.0
Chronic conditions = yes, by age group								
2–6	95	6.1	443	3.3	14	1.6	312	1.0
7–17	257	16.6	382	2.8	47	5.2	261	0.8
18–30	285	18.4	1,345	10.0	66	7.3	1,468	4.7
31–44	224	14.5	1,815	13.4	62	6.9	1,815	5.8
45–64	325	21.0	3,542	26.2	146	16.2	5,738	18.2
≥ 65	361	23.3	5,982	44.3	564	62.7	21,906	69.5
Total	1,547	100.0	13,509	100.0	899	100.0	31,500	100.0
Vaccination status^b^								
No	5,095	80.8	19,802	60.7	623	45.0	20,900	42.7
Yes	1,213	19.2	12,796	39.3	760	55.0	27,994	57.3
Total	6,308	100.0	32,598	100.0	1,383	100.0	48,894	100.0

^a^ Diabetes, adiposity, neurological diseases, kidney diseases, haematological cancers, cardiovascular diseases, respiratory diseases, immune diseases.

^b^ Individuals were considered vaccinated if they had received the 2021/22 influenza vaccine at least 14 days before being tested. Children between 2–6 years of age were offered the live attenuated influenza vaccine, of which 92% of vaccinated children received. The rest of the population was offered quadrivalent inactivated influenza vaccines.

Of vaccinated children aged 2–6 years, 92% (707/767) received a LAIV. The estimated influenza A vaccine effectiveness was 62.7% (95% CI: 10.9–84.4) in hospitalised children and 64.2% in non-hospitalised children (95% CI: 50.5–74.1) (Table 2). In those aged 7–44 years vaccinated with a QIV, non-hospitalised patients had a lower influenza VE of 24.8% (95% CI: 12.8–35.2). However, in hospitalised patients of the same age range, and among hospitalised and non-hospitalised patients 45 years and older, the estimated VE’s were not significant, i.e. 19.3% (95% CI: −9.9 to 40.7), −23.5% (95% CI: −44.6 to −4.9) and −5.1% (95%CI: −19.5 to 7.5), respectively (Table 2).

**Table 2:** Adjusted interim seasonal vaccine effectiveness against laboratory-confirmed influenza A in non-hospitalised and hospitalised patients by age group, Denmark, 1 December 2021–25 March 2022 (n = 89,183)

Patient setting	Age group (years)	Cases			Controls			Adjusted VE	
		All	Vacc.	%	All	Vacc.	%		
Non-hospitalised	2–6	473	62	13.1	1,692	450	26.6	64.2	50.5 to 74.1
	7–44	4,341	331	7.6	13,619	2,221	16.3	24.8	12.8 to 35.2
	≥ 45	1,494	820	54.9	17,287	10,125	58.6	−5.1	−19.5 to 7.5
Hospitalised	2–6	43	7	16.3	869	248	28.5	62.7	10.9 to 84.4
	7–44	393	63	16.0	9,312	2,109	22.6	19.3	−9.9 to 40.7
	≥ 45	947	690	72.9	38,713	25,637	66.2	−23.5	−44.6 to −4.9

Vacc.: vaccinated; VE: vaccine effectiveness.

Children between 2–6 years of age were offered the live attenuated influenza vaccine, of which 92% of vaccinated children received. The rest of the population was offered quadrivalent inactivated influenza vaccines.

Cases are patients who tested positive for influenza A virus. Controls are patients who tested negative for any influenza virus.

From week 40 2021 to week 12 2022, 1,299 of the 1,343 subtyped influenza viruses were of the A(H3N2) subtype, nine were A(H1N1)pdm09 and 35 were influenza B viruses. Approximately 10% of the subtyped virus were genetically characterised. Of 129 genetically characterised viruses, 4 were A(H1N1) and 125 belonged to A(H3N2) and of these, 124 belonged to clade 3C.2a1b.2a.2. Antigenic characterisation by haemagglutination inhibition test also revealed an 8 to 32-fold lower reaction to the vaccine strain A/Cambodia/e0826360/2020 (H3N2)-like virus.

## Discussion

In Denmark, almost all COVID-19 interventions and restrictions were lifted by the end of January 2022 (end of week 4), which might explain the sharp increase in the influenza transmission from week 6 in 2022 and onwards. The low to no effect of the administered vaccines in both hospitalised and non-hospitalised patients aged 7 years and above against the circulating influenza A stain is similar to findings in a study from the United States where the overall VE against medically attended outpatients with influenza A(H3N2) virus was estimated to 16% (95% CI: −16 to 39) [5]. In Denmark, the majority of the influenza vaccines were administered during October 2021, which was almost 5 months before the influenza A(H3N2)-dominated increase was observed. In particular, the VE against influenza A(H3N2) declines with an increase in time since vaccination [6], which could be one explanation for the generally low VE we observed in the age groups from 7 years and above. The difference between the circulating strain and the vaccine strain is another contributing factor as the circulating influenza A(H3N2) strain belonged mainly to clade 3C.2a1b.2a.2, which has changes in the antigenic sites compared with the vaccine strain.

The higher VE in children 2–6 years might be attributed to the use of a LAIV, which can result in a better immune response in the mucosal epithelia [7]. The majority of children aged 2–6 years received the influenza vaccination for the first time this season, thus two doses were administered compared with the single dose administration of the QIV in the older age groups. In addition, the LAIV is only offered to healthy children, which might also explain the higher VE compared with the risk groups with an expected weakened immune response.

This study also has limitations. Symptom onset is not registered in the national registers, this might imply that some patients testing negative for influenza might previously have been positive.

## Conclusion

In conclusion, this study showed a very late increase in the seasonal influenza transmission and no observable VE against the circulating influenza A(H3N2) strain following vaccination with the QIV in adults above 45 years, which may be due to a combination of waning immunity and antigenic drift. The study highlights that 2 years of varying COVID-19 restrictions may have affected the transmission dynamics of influenza virus. Thus, it is important to have an all-year surveillance to capture out-of-season influenza and circulation of other respiratory viruses.
